# Environmental Cognition and Mental Health: Physical Activity and Place Attachment as Pathways

**DOI:** 10.1093/geroni/igab046.1810

**Published:** 2021-12-17

**Authors:** Yingqi Guo, Shiyu Lu, Yuqi Liu, On Fung Chan, Hiu Kwan Chui, Terry Y S Lum

**Affiliations:** The University of Hong Kong, Hong Kong, Hong Kong

## Abstract

Few studies have explored the underlying pathways between environment cognition (i.e., perception of environment) and mental health in older adults. We tested the mediation effects of physical activity and place attachment in the relationship between environmental cognition and mental health, based on a survey study of 1,553 older adults in Hong Kong using structural equation model. The results showed that significant relationship between negative environmental cognition (i.e., residing in higher accessible area but perceive lower) on access to convenient stores, leisure facilities, clinics, community centers, religious places and lower mental health can be explained by lower daily average physical activity time. Place attachment can significantly mediate the positive effect of positive environmental cognition (i.e., residing in lower accessible area but perceive higher) towards all types of services on mental health. Findings from this study have policy implication for urban planning and age-friendly community design

